# Saying yes to mess: disorganization as an antecedent to dynamic capabilities

**DOI:** 10.1007/s11846-022-00540-w

**Published:** 2022-03-26

**Authors:** Dinuka B. Herath, Shelley Harrington

**Affiliations:** CS3/13, Charles Sikes Building, Business School, The University of Huddersfield, HD1 3DH Huddersfield, UK

**Keywords:** Disorganization, Dynamic capabilities, Micro-foundations

## Abstract

Modern businesses constantly operate in an environment filled with unpredictability. Such unpredictability and heightened dynamism require rapid responses, mainly prompted by the realization that such irregular phenomena cannot be dealt with using conventionally structured and organized approaches alone. In line with this ongoing conversation fuelled notably by the COVID-19 pandemic and its associated digital acceleration, in this paper, we lay out the case for how “*actively embracing disorganization*” as a new work form can be a mechanism that facilitates the creation and enactment of important dynamic capabilities thus enhancing a firm’s ability to cope with unpredictable environments. In doing so, we attempt to make a novel contribution outlining the relationship between disorganization and dynamic capabilities through propositions and an accompanying conceptual framework. In this article, we position disorganization as a form of organizational design capable of enabling the link between dynamic capabilities and performance.

**Supplementary Information:**

The online version contains supplementary material available at 10.1007/s11846-022-00540-w.

## Introduction

The volatility of the modern-day business environment creates unease among workers, managers, and organizations alike (Frank, Güttel & Kessler, 2017). Such volatility has been further heightened as a result of the pandemic which has triggered new forms of working (McAfee et al., 2012) and stimulated an acceleration of technological advancement. These complexities invariably lead to a high rate of business mortality (Ciminelli & Garcia-Mandico, 2020). One of the root causes of this increase in organizational mortality, we argue, is the inability of organizations and managers alike to adapt to these highly demanding complexities (Reeves & Deimler, 2011).

One of the strategies utilized by modern-day organizations in dealing with complexities is to harness the strategic power of dynamic capabilities (Teece et al. 2016). Dynamic capabilities are those capabilities that an organization needs to sustain competitive advantage within complex environments (Teece 2012). The body of knowledge pertaining to dynamic capabilities is rich and well developed. However, there are open questions (Peteraf, Di Stefano & Verona, 2013) relating to how to strategically design organizations in order to foster dynamic capabilities. In this paper we present the case for disorganization as one such strategy, thus extending the conversation started by Felin and Powell (2016). Disorganization is the process of breaking down restrictive organizational structures and routines that commonly reside in organizations in order to increase organizational performance (Herath 2017). Here, we argue, the very state of disorganization is a state that should be encouraged to develop dynamic capabilities needed not only to deal with the complexities of the modern business environment but also to sustain competitive success. Dynamic capabilities, by their nature, are underpinned by flexibility and fluidity and yet such conditions are often not truly fostered within an organization where boundaries and restrictions are placed on organizational life.

Grounded in the dynamic capabilities perspective, our paper addresses how dynamic capabilities can be developed through a focus on disorganizing those structures that often underpin them. Prior studies, emerging from the dynamic capabilities literature, have focused attention on the micro-foundations of dynamic capabilities (Teece, 2007; Felin et al. 2012) and yet they have often neglected the wider, more organizational structural/practice elements that we deem important. As such, we propose that the neglected and novel role of disorganization is an important antecedent and one that focuses on further unpacking the black box of dynamic capabilities by offering a more practically orientated approach to understanding and then enabling the conditions needed to establish their presence.

This paper directly responds to calls from Teece (2018: 47), who discusses the need for research that focuses on “specific aspects of dynamic capabilities”, which he notes includes a focus on “flexibility to illuminate aspects of business model innovation and implementation” thus reinforcing a less stable framework of dynamic capabilities (Teece, 2007). Here, we contribute by unpacking the ways in which disorganization stimulates flexibility, in turn strengthening dynamic capabilities and their relationship with performance. Sticking to highly organized and formalized procedures might restrain creativity in solving non-recurrent problems (Adler 1999). Rules and routines might be useful for dealing with structured and simple problems or executing operations with efficiency. However, they do not help managers to understand the causes behind the cause of action (Pertusa-Ortega et al. 2010) and deploy dynamic capabilities. In turn, this emphasizes the more “entrepreneurial and less routinized” aspects of decision making required to drive dynamic capability creation (Teece, 2007; Augier and Teece 2008).

In order to make the aforementioned contributions, the paper is structured as follows. First, we provide the theoretical justification for the relationship between disorganization and dynamic capabilities. In doing so, we provide conceptual clarification of the concept of disorganization in relation to similar concepts of agility, flexibility, ambidexterity, chaos, and complexity. Second, we present a set of propositions along with an accompanying conceptual framework outlining this theoretical link between disorganization and dynamic capabilities.

## Theoretical Framework

### Disorganization

Over the years, firms have become extremely adept at “organizing”; however, no matter how well the “organizing” is carried out, things rarely go exactly to plan. Organizing also carries costs (tangible and intangible) and imposes pressures on organizational units (Herath 2019) and individuals (Crozier 1969), which grow rapidly as the level of organization required increases. Therefore, as research suggests, firms cannot just organize their way out of every problem, regardless of how sophisticated the organizing process is, thus embracing continuous change processes (Rindova & Kotha, 2001). The question then becomes, “How comfortable are firms in the face of changing plans, randomness, and dynamism?” Could we design firms that are resilient, anti-fragile, and which have adequate malleability to incorporate the inherent disorganization present in modern businesses and their environment? The modern concept of “disorganization” in management thinking tries to systematically provide the answers to these questions. In this paper, the focus is not on coming up with progressively cleverer ways of “organizing” per se; instead, it is on emphasizing the importance of embracing and leveraging disorganization as a viable strategic option.

The concept of disorganization was first introduced into the management lexicon in the 1960s (Beardsley 1968; Crozier 1969). Unlike most other concepts, disorganization was not initially developed in order to improve organizations. Rather, it was a concept that emerged from observations within organizations (Cohen & Levinthal, 1990; Desouza and Hensgen 2015). Disorganization can be defined as the “*stochastic accumulation of entities within hierarchically ordered complex human structures*” (Abrahamson 2002: 139). Stochastic accumulation refers to the unpredictable and unplanned accumulation of entities within a system. The entities could be both physical (random accumulation of papers on a desk, filing cabinets) and non-physical (relationships, information structures, group dynamics, and databases) aspects of an organization.

Disorganization can be observed in all organizations, regardless of other factors (such as sector or type of operation). The stochastic accumulation of entities also occurs in every hierarchical level within a given organization (the mailroom or the boardroom) and can be studied from multiple vantage points (micro, meso, or macro lenses). In such cases, the entities themselves might differ but the stochastic, unpredictable, unplanned (non-teleological) element of such accumulation is ever present.

The coping strategies initially utilized by practitioners when dealing with disorganization have largely been based on fear (uncertainty avoidance). One of the primary reasons for such a reactive approach to disorganization is largely related to the apparent assumption held by many managers that in order to increase productivity, high levels of organization (order) are needed (Amabile 1983; Weick 1998; Ashmos et al. 2002). Therefore, any disorganization is assumed to be detrimental by default (Knights and Vurdubakis 2005). However, Crozier (1969) and Abrahamson (2002) argue that, contrary to popular belief, such an assumption is unwarranted and that over-emphasizing organizing can have detrimental effects on productivity and employee well-being within organizations. For example, some organizations with well-structured and formalized communication channels might have some problems to react in the face of change, since signals of the need for change need to filter their way to decision makers and back again to those entrusted with execution (Child & McGrath, 2001).

Furthermore, over-emphasis on organizing all elements within organizations creates unnecessary pressures, which cannot be justified by the benefits that such processes yield. Recent developments in attempts to highly organize all aspects of work have reiterated the perils of over-reliance on organization (Nonaka, 1988; Reeves, 2016). As such, the steadily growing complexities, both within and outside organizations, in the twenty-first century require a new approach to dealing with disorganization.

**Fig. 1:**
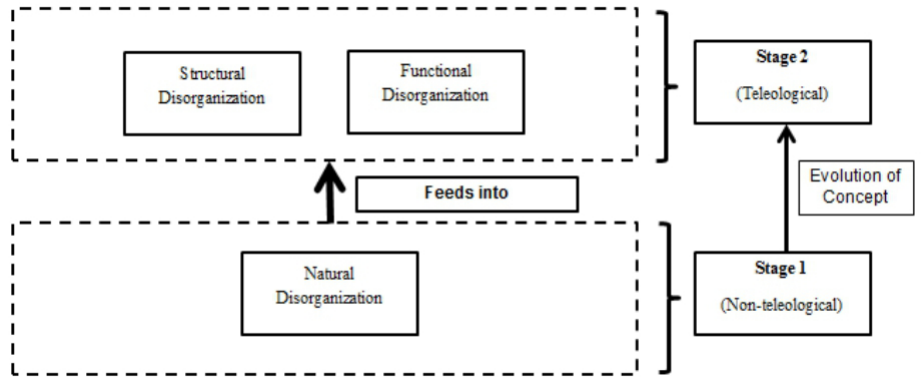
Evolution of disorganization

Recently, the concept of disorganization has received conceptual clarification and systematic attempts are being made to study the phenomenon using multiple methods (Alvesson and Spicer 2012). Under this new wave of research interest, the reactive approach to dealing with disorganization (we coin as stage 1) has been transformed into a more proactive approach – stage 2 (Haford, 2017). The difference between the two approaches lies in the intentionality and planning involved in handling disorganization. In the reactive approach (stage 1) disorganization was seen as naturally occurring without any intervention or planning involved (non-teleological), as such was seen as unpredictable and unmanageable (Abrahamson 2002). In contrast, the proactive approach (stage 2) looks at the specific structural/functional design levers and mechanisms to plan for, control, and enact disorganization in firms to gain full effect; as such stage 2 introduces a teleological (goal driven) approach to the phenomenon (Herath et al. 2017). This new approach to disorganization shifts the focus from mere coping strategies to the study of how disorganization can be leveraged for specific organizational goals (Abrahamson 2002). In this vein, recent studies have looked at how disorganization can be used for goal-setting and improving employee motivation (Herath et al. 2017). Further attempts have been made to look at how disorganization can be leveraged in order to increase problem-solving efficiency (Fioretti and Lomi 2010). Emergent solutions and the juxtaposition of unconventional entities to enable faster problem-solving are among the other benefits identified (Warglien and Masuch, 1996; Janseen et al., 2016). More recent studies have looked at disorganization as an enabler/enhancer for organizational adaptability (Herath et al. 2017). Motivated by the advantages that disorganization is postulated to provide, recent work on the topic has explored combining stage-1 (reactive) and stage-2 (proactive) disorganization in order to further maximize its utility in modern organizations. A summary of these developments is presented in Fig. 1.

Figure 1 depicts the conceptual framework of how disorganization as a concept has taken shape. Structural disorganization refers to disorganization that can be introduced into the structures of an organization. From a macroscopic point of view, the structures refer to the large-scale topology of the organization, such as hierarchical structure, power structure, and information structure (Li and Tan 2013; Herath 2019). Using a microscopic lens, the structure refers to the topology of a team or department. Making such structures flexible by reducing structural barriers, enabling stochastic accumulation of entities (natural disorganization), and reducing the amount of effort to reorganize the structure refer to structural disorganization. The parameters (level of flexibility, number of barriers reduced, rate of accumulation of entities, and amount of effort to reorganize) can be varied depending on the organization and its needs. Furthermore, the BDVI (breadth, depth, volume, and intensity) of such structural disorganization can also be varied to achieve the desired level of disorganization within the system. Functional disorganization (b) refers to the functional agility within the structures of the organization. Therefore, on a macroscopic scale, such disorganization focuses on the routines and procedures in operating within the organization to carry out tasks (i.e. access resources, make relationships). Using a microscopic lens the focus shifts to the routines and processes within and among teams and individuals. As with structural disorganization, functional disorganization can also be varied in terms of parameters (number of barriers in accessing resources, ease of access to quality resources, and the range of maneuverability of individuals/teams). If both structural and functional disorganization were combined, an organization could become highly adaptable and rapidly respond to internal and external stochasticity in a proactive manner (Secchi 2019).

### Dynamic Capabilities

Dynamic capabilities, defined as “the firm’s ability to integrate, build, and reconfigure internal and external competencies to address rapidly changing environments” (Teece et al. 1997: 516). The notion of environmental dynamism, by its very nature, denotes change and, while, as argued by Winter (2003), there are many ways to change, the study of dynamic capabilities argues that the more sustainable, competitively oriented forms of change are those underpinned by the presence of dynamic capabilities. This is further supported and driven by the strong links evident between dynamic capabilities, firm survival (Vrontis et al., 2020), and firm performance (Teece 2014; Wang et al. 2015).

Forming the theoretical framework for the micro-foundations approach to dynamic capabilities, Teece (2007) disaggregated them into three activities: (1) to sense opportunities – *sensing*; (2) to seize identified opportunities – *seizing;* and (3) to maintain competitiveness through the transformation of ordinary capabilities – *transforming.* The ability to undertake the three activities of sensing, seizing, and transforming is not perceived to be uniformly distributed across individuals; nor are the activities considered to be entirely sequential (Teece et al. 2016). As a result, there is a need to understand how to design organizations in a way that enables the development and enactment of dynamic capabilities in a non-routinized manner thus highlighting the role of the individual. The topic of organizational design for dynamic capabilities has been addressed by Felin and Powell (2016), who put forward design principles including polyarchy, social proof, and open organization as a way of encouraging the achievement of dynamic capabilities within the firm. Extending this work, disorganization is positioned as a specific type of open organization, which can be practically engineered to support dynamic capability development.

Teece et al. (1997) position dynamic capabilities as processes that are shaped by positions and paths, with Felin and Powell (2016) extending this to include organizational design. This is further explained in the work of Helfat et al. (2009) and Maritan (2007), who depict processes and paths as the mechanisms by which dynamic capabilities come into play. At the process level, attention is directed to the internal position of the firm, namely, market value, structural assets, and the external position of the firm, including how the firm interacts with its institutional environment and market forces. Teece et al. (1997) argue that a firm’s position in this vein influences the ability of a firm to gain competitive advantage, reinforced in part by the uniqueness of this position (Barney 1991). Paths refer to the history of the firm and, as Teece et al. (1997: 522) state, “bygones are rarely bygones”. This infers that a firm is shaped by the past, present, and future.

#### Internal Factors

The key role that managers play in the firm’s ability to adapt is well explored across the extant literature with the entrepreneurial capabilities school of thought arguing that entrepreneurial, flexible thinking plays a key role here (Augier and Teece 2008). Teece (2007: 1346) notes that “dynamic capabilities reside in large measure with the enterprise’s top management team” but, because of path dependency, these dynamic capabilities are impacted by the organizational processes, systems, and structures that the enterprise has created to manage its business in the past. The emphasis on organizational structure is further explored in the work of Song et al. (2016), who identify an organizational structure that they argue supports internal co-opetition, as well as being an enabler of dynamic capabilities. Lawson and Samson (2001) also argue that organizational structure should be harnessed to support innovative practices within the firm. They argue that structure needs to reflect the fluid and creative basis of innovative practice, much like what is offered through structural disorganization. They further argue that a move toward formalized structures has created a programming of mainstream business capability where there is no encouragement to think outside the box. This reinforces the thinking of the co-founder of Hewlett Packard, who stated that “the creative process works well when it is not too structured” (Platt 1997: 5). A modern conceptualization of this, we propose, would be to position disorganization as a way of enabling the development of innovation capabilities within the firm; for example, transforming requires a commitment to innovative capability development and reconfiguration to move forward. High-performing firms are firms where we see high dynamic capabilities; we argue that they take a step back from mechanistic and institutionalized bureaucracy and instead create permeable business boundaries to break down barriers in order to create a non-routinized, flexible, and more entrepreneurial approach to the development of dynamic capabilities.

Disorganized work environments “inevitably lead to increased levels of employee autonomy” (Homberg 2019: 118), which in turn drives creativity. Examples of organizations that have already experimented with disorganization include IDEO, a Silicon Valley design firm that has engineered a number of re-design processes to enable heightened creativity, and Oticon, which, as noted in DeFillippi and Lehrer (2011), has made its entire firm project-based. Such examples, as noted by Homberg (2019: 118), act as an “exemplification of the powerful forces disorganization techniques can release”.

Pablo et al (2007) argue that there is a need to identify, enable and manage dynamic capabilities. Eisenhardt and Martin (2000: 1005) propose how enablers “facilitate or provide the catalyst that initiates the engagement of dynamic capabilities”. For example, they refer to the importance of cross-functional teams. This draws on a suggestion across the field that the effective engagement of dynamic capabilities must be “contingent on first engaging certain enablers suitable to changing environmental conditions” (Walshe et al. 2010: 255). However, to date, the field has been relatively silent about the crucial role of enablers.

### Disorganization in Practice

Having explored both the concept of disorganization and its applicability to dynamic capability development, the ways in which this relationship is to be instantiated in a firm have to be considered. It should be noted that leveraging disorganization or using disorganization within organizational structures does not mean “*organizing*” the disorganization process. Instead, the focus is on where, when, and how disorganization should be tolerated or initiated. Thus, the stochastic accumulation of varied entities that the definition of disorganization entails remains intact. Determining the best place to use such a process does not negate its stochastic accumulation. Once the location has been selected, the process of disorganization carries on, as its definition suggests. Thus, it is important to explore what specific organizational interventions enable the enactment of disorganization.

In the extant literature, such interventions have two major thrusts: (a) strategic intervention (Harford 2017); and (b) disorganization-specific organizational policy (Abrahamson and Freedman 2013). Both these components are closely related, such that, in order for the disorganization-specific policies to be implemented, the organization first has to make a strategic decision to embrace disorganization (Jansen et al. 2017). Here, embracing means setting up the necessary conditions (in terms of resources and policy framework) for the successful enactment of disorganization in a firm (not organizing the disorganization, but setting the conditions to make disorganization happen). In this regard, three general interventions could be enacted within a firm to leverage natural, structural, and functional disorganization.

**Table 1:** Mechanisms of implementation

Type	Corresponding firm-level policy
*Natural*	Creating an environment where conventionally isolated or non-interacting entities can routinely engage in tasks related to sensing, seizing, and transforming. This is akin to assembling a team comprising of experts with heterogeneous expertise which rarely interact under standard operating procedures. This enables novel ideas to emerge and fosters dynamic multifaceted relationships that are capable of dealing with the dynamism.
*Structural*	Providing structural flexibility to organizational units and/or individuals to seek resources across structural boundaries. Here structural boundaries are hierarchical levels, departmental and team confines. For instance, when a team is engaged in sensing, seizing, or transforming, they can be afforded access to resources without conventional structural limitations thus enabling an accumulation of relevant entities at a higher rate.
*Functional*	Providing procedural flexibility to organizational units and/or individuals to seek resources across functional boundaries. This is closely related to the structural element with the difference being the focus on procedures involved in resource seeking. Reducing or eliminating procedures (reducing the number of steps in collecting resources needed for completing a given task) and increasing the range of operations a given individual or team can perform is thus the act of increasing functional disorganization.

The interventions in Table 1 can be seen as the most rudimentary instantiation of disorganization in a firm in order to enhance dynamic capability development. More sophisticated approaches can thus be derived from the foundational interventions outlined here. Deriving from past examples of such implementation, Harford (2017), Secchi (2019), Herath (2019), and Homberg (2019) have demonstrated how such interventions can be implemented. For instance, the enactment of these interventions can be carried out simultaneously or in a sequential manner (where one policy is enacted after the other). Likewise, the location of the policies’ enactment, while most suitable at the firm level (given the strong influence of firm-level decisions on employees), can also be instated at lower levels (i.e. department or team-specific policies). With these policies in mind, the boundary conditions of these interventions have to be considered. Clearly, not every organizational setting requires disorganization (i.e. an assembly line). However, even in a highly structured organizational setting, having some acceptance of disorganization (albeit to a limited degree) reduces the focus of a firm on organizing. These interventions are especially suitable for organizational settings that require creative and new idea generation (Abrahamson and Freedman 2013). For instance, even in a firm with assembly lines, the design and idea generation for new products require the participation of knowledge workers, who, as research shows, require a significant level of autonomy (Crozier 1969). Thus, while disorganization is indeed more useful in firms that have knowledge workers undertaking complex tasks (Homberg 2019), it is still useful in highly structured settings, albeit to a lesser degree.

## Propositions

### Disorganization, Dynamic Capabilities, and Performance

Teece et al. (1997: 511) propose that the role of dynamic capabilities is to equip the firm with an improved positioning to deal with heightened environmental dynamism and volatility within the external environment, and thus dynamic capabilities “directly fight against environmental change”. While in a stable, core environment, the development of ordinary capabilities is central to survival, within rapidly changing environments dynamic capabilities emerge as a powerful weapon of survival. In line with this, disorganization can be utilized as a mechanism for enhancing rapid organizational adaptability and the creation of new knowledge. In such conditions, disorganization can be used as a leveraging process to enable such adaptability.

An organization utilizing disorganization in the dynamic capability development process will have the opportunity to use disorganization proactively. To this end, disorganization can be introduced as a mechanism for removing both structural and functional barriers among stakeholders. From a structural standpoint, this process involves purposefully designed changes to the hierarchical arrangement and rules of control in teams to facilitate or improve the accumulation of entities within them (Abrahamson and Freedman 2013). This involves, offering structural flexibility to organizational teams and/or individuals to pursue resources across structural boundaries. Here structural boundaries are hierarchical levels, departmental, and team boundaries. For example, *in a low-performing team with an inflexible chain of command, modifying the decision-making process to a more organic, democratic setup.* From a functional standpoint, the process involves purposely designed adjustment of the rules of interaction (socialization constraints) imposed on individuals and teams when pursuing resources to facilitate or improve the accumulation of entities. This involves, offering functional (procedural) plasticity (Homberg 2019; Siebers et al. 2021) to organizational teams and/or individuals to seek out resources across functional boundaries. Thus, decreasing or removing procedures (i.e., decreasing the number of steps in accumulating resources necessary for accomplishing a given task) and enhancing the range of operations a given team can execute (Harford 2017; Herath 2019). For example, *giving autonomy to a team member to deviate from their routine patterns of work where needed or providing members with the autonomy to decide to self-organize their work patterns*.

These interventions geared towards fostering disorganization then contribute to the enhancement of the sensing, seizing, and transforming processes in dynamic capability development given they boost organic interactions among relevant actors (Holland 2006; Siebers et al. 2021), increase resource-seeking scope given the less structural and functional obstacles (Herath et al. 2016) that in turn enhances collaboration in business units (Homberg 2019) along with enabling top management teams to respond much faster to environmental dynamism. Recent empirical work (Herath 2021) that used UK Workplace Employment Relations survey data examining the efficacy of disorganization on employee performance, further corroborates the utility of disorganization in enhancing various organizational processes as discussed here. Furthermore, some examples of applications of this sort can be seen in the cases of MIT Building 20 (Howland 2014) that generated extraordinary results in an infamously unstructured environment (Krakauer et al. 2019) along with cases such as Oticon (Foss 2003) with their Spaghetti organizing approached that incorporated both structural and functional interventions discussed above (Homberg 2019).

When considering the sensing process, research shows that in dealing with often complex and contradictory information, organic flexible working conditions are preferred (Russell et al. 2009). Such pockets of flexibility, however, can be induced in organized structures (Brodbeck 2002). Disorganization, as characterized here, can be used for such a purpose that it can be used as a mechanism for enabling collaboration. Such a process can be made simple or sophisticated, depending on the nature of the problem (Abrahamson 2002). However, as a basic process, reducing hierarchical power structures and unnecessary authority in a team can enable such plasticity in the sensing process. The sensing process itself tends to deal with complex information that is often stochastic and contradictory in nature (Schilke 2014). The team involved would then have the structural and functional flexibility to access resources and make the necessary relationships in the sensing process.

The seizing process requires the knowledge generated through the sensing process to be utilized when addressing the opportunities uncovered in the marketplace (Katkalo et al. 2010). This process is therefore inherently innovative and requires moving beyond the conventional thinking when seizing opportunities presented (Kindström et al. 2013). Here too disorganization plays a role in reducing structural and functional barriers within the seizing process. It could be argued that the highest utility of using disorganization in developing dynamic capabilities can be seen in seizing and transforming processes. Both seizing and transforming require going beyond the current state of affairs and coming up with novel and transformative ideas. Such a process is labor-intensive and requires a degree of innovation (Breznik and Lahovnik 2014). As Abrahamson (2002) states, disorganization is an ideal candidate for creating such emergent solutions and provides the capability to juxtapose seemingly (conventionally) unrelated ideas or entities. Therefore, using disorganization to enhance the plasticity within sensing, seizing & transforming processes would be the best-suited utilization of disorganization.

The transforming process reflects the continual renewal of the organization. Transforming which is strongly linked to innovation enables an organization to examine its resource base to move it towards the intended future state of renewal and adaptation (Teece, 2007).

Even though the sensing, seizing, and transforming (Castiaux 2012) processes differ in their functionality, how disorganization can be utilized in all three processes is identical. However, the amount of disorganization in each process, and the level of disorganization in terms of breadth, depth, volume, and intensity, can be varied according to the nature of the problem. Studies show that disorganization provides efficiency advantages when dealing with both simple and complex problems (Herath et al. 2016). However, the effect of disorganization in dealing with complex problems is more pronounced than when dealing with simple problems (Herath et al. 2017). This is due to the complex nature of the resources required and the relationships needed for such complex problems to be solved (Abrahamson and Freedman 2013). Reducing structural and functional barriers through disorganization, therefore, enables the individual or team to traverse a larger range of possibilities, seek resources, make relationships and obtain resources in a more efficient manner. Therefore disorganization is a process, which provides a mechanism both to deal with stochastic elements in the dynamic capability development process and to engender rapid adaptability within the processes of sensing, seizing, and transforming. Based on this discussion, we, therefore, propose that disorganization positively influences the relationship between dynamic capabilities and performance.

#### Proposition 1

*Disorganization enhances the generation of dynamic capabilities*.

### Non-Routine Tasks in Disorganized Environments

The entrepreneurial function embedded within dynamic capability creation highlights the less-routinized, more flexible aspects of decision making (Augier and Teece 2008). This differs to those who argue for the more stable, routinized aspect of dynamic capability creation (Helfat and Martin 2015).

While routine is required in part and, may differ depending on the firm, an imbalance between routine and capability development could result in the occurrence or reinforcement of organizational inertia and thus restrict dynamic managerial capabilities where more fluid processes of innovation and knowledge-sharing are required (e.g. Kindström et al. 2013; Pérez-Valls et al., 2019). As such, Teece (2017) argues that “in times of turbulence, routines can be dangerous” (p. 116). We propose that when disorganization is welcomed and allowed to reside within the organization it encourages entrepreneurial actions within the organization, which can only be routinized up to a point thus supporting the contention that individuals may drive, through their entrepreneurial action, certain dynamic capabilities (Teece 2012). We argue here that disorganization facilitates the adaptation of routines within the organization in response to environmental changes which in turn drives dynamic capability creation. Explicit efforts are required to modify existing routines and we argue there is a need to actively de-routinize in order to support Zollo and Winter’s (2002) argument that dynamic capability creation must “invoke mechanisms that go beyond semi-automatic, stimulus-response processes’ (p. 13). Disorganization and the breaking down of routine structure facilitates a more proactive, flexible structure which therefore highlights the value of disorganization as an input to the creation of dynamic capabilities through the evoking of mechanisms that support the evolutionary, fluid actions of individuals as opposed to the structured, response processes often seen.

Moving away from the routinized environment of traditional organizations and the structured, persistent nature of dynamic capabilities, disorganization creates an opportunity for entrepreneurial managers to excel at sensing, seizing, and transforming in a manner that is highly creative and fluid and thus not restricted by boundary conditions. This is achieved through a conscious, pro-active effort to de-structure routines and to move away from replication and routine which may limit the creative, flexible evolution of dynamic capability creation.

This, in turn, responds to a criticism of the current thinking within the dynamic capabilities framework, where it is viewed as being bound by external conditions (Feng et al. 2017). Routinizing action in such environments becomes both difficult and unrealistic in highly volatile environments. Excelling thus becomes central, as the ability to excel is more pronounced when restrictive, time-slowing conditions are removed (slow channels of communication, top-down restrictive organizational structures, and bureaucracy).

The extant literature in the field of dynamic capabilities has focused on the role the top management team plays in the development and enactment of dynamic capabilities (e.g. Teece, 2007; Kor and Mesko 2013). Teece (2007) highlights that sensing “must be performed by the top management team” and thus acknowledges their importance (p. 1319). If importance is placed at this level, then we propose that, within highly dynamic, volatile environments the organization naturally has to rely on the entrepreneurial thinking and actions of the top management team, which do not have time to become embedded or routinized within the organization thus limiting the structured, persistent nature of dynamic capabilities. The degree of instability that emerges is therefore powerful for progression and for innovative thought to develop. Building on this we propose that a decrease in the need for the firm’s routinization increases the effect of disorganization on the relationship between dynamic capability development and firm performance. Therefore, routinization acts to moderate the effect of disorganization such that when the routinization in a firm decreases the effect of disorganization increases.

#### Proposition 2

*The level of routinization negatively influences the effect of disorganization on dynamic capabilities*.

### The Relationship between Disorganization and Environmental Dynamism

Disorganization is seen as a naturally occurring phenomenon, which, if embraced and leveraged, can be used to maximize individual, team, or organizational functions (Abrahamson and Freedman 2013). While the proposed benefits of disorganization are well articulated in terms of dynamic environments, its utility is discussed less in highly stable environments (Stacey 1992). Conventionally, such stable environments, and the firms that operate in them, have seen disorganization as a detrimental phenomenon. If the environmental dynamics are stable, enacting disorganization actively in a firm does not have a clear purpose (Blau and Schoenherr 1971), as such firms do not regularly require dynamic responses (Becker 2004). Levinthal and Marino (2015) add to this point by showing that when the environment is stable and the responses required are highly routinary, disorganization does not provide a significant improvement to conventional structured methods. In fact, if a firm has a stable response to a particular problem crystallized over years of calibration, disorganizing such a process could create instabilities to long-established solutions. Thus, if the cost of disorganizing is higher than the benefit it yields, that process should not be disorganized. This does not, however, account for the natural forms of disorganization that occur even in the most stable firms (Herath et al. 2017). However, it is a convincing argument against actively leveraging disorganization in situations where dynamism is not required.

In contrast, when looking at environmental dynamism more generally the same argument can be mounted in favor of disorganization as the level of environmental dynamism increases (Stieglitz et al. 2016). When a given entity (physical or non-physical) is in a dynamic disorganized state, if the process of organizing the entity costs more than the benefit that such a process yields it should equally be questioned whether that entity needs to be organized in the first place (Abrahamson 2002). Research shows that organizing processes without clear accountability for the benefits of such an organizing process leads to wastage, in both monetary and manpower terms. Furthermore, the frequency of organization also falls prey to the same argument. If the frequency of organizing an entity yields less benefit than the organizing iterations that are required, it is logical to question the viability of such a process. Organizing for the sake of organizing therefore is counterproductive, as much as disorganizing for the sake of disorganizing is for effective performance within organizations (Alvesson and Spicer 2012). However, there are a few counter-arguments against such a cost-benefit-analysis approach to organizing/disorganizing processes (Landy et al. 2017). The primary rebuttal is based on the fact that when analyzing the benefit of the organizing processes people tend to focus on tangible improvements only, and intangible benefits are largely ignored. For example, organizing a messy desk over and over again might not be efficient in terms of manpower; however, the psychological satisfaction that the end result yields in terms of a clean desk could outweigh the other benefits/costs that the process produces. This line of reasoning, however, is primarily suited when considering the organizing processes undertaken by single individuals. Furthermore, there is no reason why such a cost-benefit analysis could not gauge intangible benefits, also given that tangible and intangible benefits are not mutually exclusive and often related (Landy et al. 2017).

Taking the environment of highly dynamic environments characterized by their instability, for example, heightened competitive environments requiring a heightened competitive response, we propose that disorganization supports an organization’s ability to deal with heightened levels of environmental dynamism to sustain competitive success as a result of the flexibility and change in attitude (related to change) that it promotes. Therefore, we propose that the utility of disorganization increases with the level of environmental dynamism, such that enacting disorganization in the process of dynamic capabilities in a highly volatile environment (Hollnagel 2017) is more useful than enacting disorganization in more stable environments. Invariably, this means that, as a consequence, we observe a very little (perhaps negligible) positive effect of disorganization in highly stable environments, which require very slow processes of response and adaptation.

#### Proposition 3

*Environmental Dynamism positively affects the influence of disorganization on dynamic capabilities*.

## Discussion

In substantiating and synthesizing the link between disorganization and dynamic capabilities presented through the propositions, the following conceptual model can be considered.

**Fig. 2:**
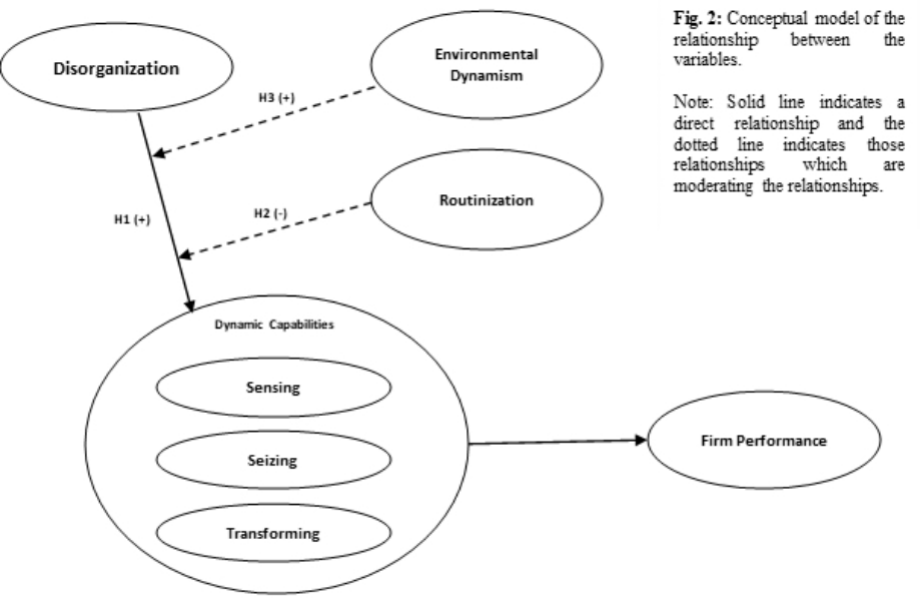
Disorganization and dynamic capabilities conceptual model

In the model (Fig. 2), H1 indicates a line of argument presented through proposition 1, where disorganization is an antecedent to dynamic capabilities. Thus, Disorganization enhances the generation of dynamic capabilities. H2 indicates proposition 2, where the level of routinization in a firm negatively influences the effect of disorganization on dynamic capabilities. Similarly, H3 indicated proposition 3, Environmental Dynamism positively affects the influence of disorganization on dynamic capabilities.

Cumulatively, the propositions substantiated through the conceptual model presented in Fig. 2 serve a specific role in crystallizing the conceptual link between disorganization and dynamic capabilities. The first proposition arguably makes the most general case, in which the capability of disorganization to generate dynamic capabilities is established. The second proposition makes a more specific case, in which the effect of disorganization is positively correlated with less routinized, more entrepreneurial aspects of dynamic capabilities. This means that, while in proposition 1 we argue for disorganization being an antecedent for dynamic capabilities in general (routine or non-routine), in the second proposition this relationship is further dissected such that there should also be a significant difference between the effects of disorganization on routine and non-routine forms of dynamic capability. The third proposition acts to qualify the other two propositions such that it specifies that when environmental dynamism is high the effect of disorganization should increase. While the conceptual model outlined focuses on firm-level measures, they can be adapted to measure team or individual-level dynamic capabilities, performance, and disorganization. Building on this conceptual model, there should also be some consideration of the measure of disorganization. In the extant literature (Herath 2021), disorganization can be measured through objective measures or perceptions (firm, team, or individual level). The perceptions of disorganization present a unique problem. This is that, conventionally, the perceptions of, and toward, disorganization have been largely negative (Abrahamson and Freedman 2013). This has mainly been due to an over-emphasis on organizing and a misconception about “what” is constituted by disorganization in a firm (Alvesson and Spicer 2012). While recent work has made progress in dispelling some of these misconceptions (Homberg 2019), it is plausible that an instance of disorganization still generally carries negative perceptions (Herath 2017; Secchi 2019). Therefore, if the measurements are carried out using firm-, team- or individual-level perceptions, the actual effect of disorganization might not easily be assessed. If the measurement is carried out in an organization in which the top management has explicitly vouched for embracing disorganization through the organizational culture, policies, and procedure, in such a case perception toward disorganization would be different to a firm in which such explicit support for disorganization was not present. Therefore, the existence, or not, of firm-level intervention and support for disorganization is a crucial consideration when measuring the phenomenon.

More broadly, in terms of implications, building on the three propositions and accompanying conceptual model discussed above, a cumulative case for the general applicability of disorganization into dynamic capability development within organizations can be mounted. This proposed relationship between disorganization and dynamic capabilities would then lead organizations to be more adaptable to external turbulence. Dynamic capabilities enhanced through disorganization thus provide firms with the security of being able to rapidly respond and adjust to novel external stimuli. Moreover, dynamic capabilities, by definition, provide a degree of adaptability to the organization. Therefore, dynamic capabilities enabled through disorganization can be seen as a mechanism to achieve even higher levels of organizational adaptability compared to conventional strategies. Therefore, we postulate that firms that embrace disorganization will be more adaptable than firms that do not embrace disorganization in the emerging and changing technological landscape.

Furthermore, the ideas presented in this article opens a few promising research directions.

**Table 2:** Future research questions

Research Questions	Explanation
Can the proposed link between disorganization and dynamic capabilities be observed in applied settings?	Each of the links made in the conceptual model (Fig. 4) can be hypothesized and investigated in future studies. ***H1***: *Disorganization enhances the generation of dynamic capabilities* ***H2***: *The level of routinization negatively influences the effect of disorganization on dynamic capabilities* ***H3***: *Environmental Dynamism positively affects the influence of disorganization on dynamic capabilities*
Further Research Questions	● Are there other effects acting on the proposed relationship between dynamic capabilities and Disorganization? ● There are multiple implementation strategies currently envisaged (Herath 2019); *which of these approaches provides the most utility?*

As depicted in Table 2, we would invite future empirical work to test the aforementioned hypotheses. In particular, this calls for a need to focus attention on the measurement of dynamic capabilities and disorganization in a way that aligns with their fluid nature but also enhances empirical applicability. We would also invite research to take place across a range of organizational settings in order to investigate how disorganization can be implemented in each of the dynamic capability development processes. Given that contextual factors play a role in developing dynamic capabilities, investigating the implementation of disorganization in sensing, seizing, and transforming in varied contexts would add value to the body of knowledge, with a particular focus on large-scale organizations.

## Concluding Remarks

This paper has drawn on a wide range of literature in order to put forward the case that disorganization is an important consideration when discussing and addressing how dynamic capabilities can be fostered within organizations through organizational design. Specifically, it proposes the value of adopting a disorganized state within the organization, and how this can encourage and facilitate a more fluid, flexible response to the external dynamics faced by firms. Disorganization in this vein is positioned as a way of openly organizing the firm in a very practically oriented manner to welcome and encourage the necessary flexibility.

There has been a strong movement, underpinned by support for the power of dynamic capabilities, toward understanding how dynamic capabilities can be achieved and how firms can ensure a competitive response despite the difficult conditions faced, and yet this remains at a black box level. This paper, therefore, tackles a very timely issue, and one that addresses a changing state of play for organizations, while suggesting that this change should not be feared but used instead as a driver of a new form of work that embraces flexible, fluid practices within the firm. Our paper provides an important contribution through the description of novel and compelling propositions, which contribute to shedding light on the black box nature of dynamic capabilities and outlining how dynamic capability development can be enhanced to meet the modern challenges firms face. Furthermore, the propositions developed, once empirically tested, may enable the prediction of dynamic capabilities within firms. This is likely to have important practical implications for how through this new form of work modern firms react to external environmental changes and how they aim to move toward a more flexible business environment capable of dealing with change. Thus, we believe the timely conceptual link proposed in this paper will help further scholarly interest in the area, prompt conversation, collaboration and invite further investigation.

## Electronic Supplementary Material

Below is the link to the electronic supplementary material.

Supplementary Material 1

## Data Availability

Not applicable.
